# UPDATING AND TESTING THE PASRR SCREEN IN KANSAS: REAL WORLD IMPLICATIONS

**DOI:** 10.1093/geroni/igz038.3403

**Published:** 2019-11-08

**Authors:** Carrie L Wendel-Hummell, Hyun Kang, Darcy Sullivan, Alan O'Neal, Lora Swartzendruber, Marion Boyd, Tracey LaPierre, John Poggio

**Affiliations:** University of Kansas, Lawrence, Kansas, United States

## Abstract

Kansas is updating the PASRR (Preadmission Screen and Resident Review) Level 1 screen per new guidance from the PASRR Technical Assistance Committee (PTAC), via a partnership between the State and university researchers. PTAC has directed states to screen for undiagnosed serious and persistent mental illness (SPMI) and also recommends screening for substance related disorders. Stakeholders were engaged through advisory workgroups and a content validity expert panel. These activities led to the creation of a revised PASRR Level-1 screen, but stakeholders also raised several concerns. PASRR law does not require Level-1 assessors to have professional training in mental health diagnoses or treatment, yet new guidelines asks them to screen for undiagnosed SPMI. Further, there are apparent discrepancies between these new guidelines and PASRR Level-2 criteria. Finally, current information management systems are not equipped to handle the higher security protocols associated substance use disorders. The draft instrument was tested with a sample of 103 nursing facility applicants by trained PASRR assessors and inter-rater reliability (IRR) was tested via a standardized vignette with 14 trained PASRR assessors. Only 3% of actual NF applicants were identified as possibly having an undiagnosed SPMI and only 43% of assessors correctly identified symptoms of a suspected SPMI in the standardized vignette, indicating poor validity and reliability in assessing for undiagnosed SPMI during the Level-1 screen. New PASRR guidelines may better ensure that nursing facility residents receive appropriate care for SPMI, however, there are many challenges to ensuring an accurate screen and supporting successful implementation.

